# How patients navigate the diagnostic ecosystem in a fragmented health system: a qualitative study from India

**DOI:** 10.1080/16549716.2017.1350452

**Published:** 2017-08-01

**Authors:** Vijayashree Yellapa, Narayanan Devadasan, Anja Krumeich, Nitika Pant Pai, Caroline Vadnais, Madhukar Pai, Nora Engel

**Affiliations:** ^a^ Institute of Public Health, Bangalore, India; ^b^ Department of Health, Ethics & Society, Research School for Public Health and Primary Care, Maastricht University, Maastricht, The Netherlands; ^c^ Division of Clinical Epidemiology, Department of Medicine, McGill University and McGill University Health Centre, Montreal, Canada; ^d^ McGill International TB Centre, Department of Epidemiology & Biostatistics, McGill University, Montreal, Canada

**Keywords:** Point-of-care, diagnosis, India, diagnostic journeys, qualitative, laboratory tests, health system, patient-centered care

## Abstract

**Background**: Depending on a country’s diagnostic infrastructure, patients and providers play different roles in ensuring that correct and timely diagnosis is made. However, little is known about the work done by patients in accessing diagnostic services and completing the ‘test and treat’ loop.

**Objective**: To address this knowledge gap, we traced the diagnostic journeys of patients with tuberculosis, diabetes, hypertension and typhoid, and examined the work they had to do to arrive at a diagnosis.

**Methods**: This paper draws on a qualitative study, which included 78 semi-structured interviews and 13 focus group discussions with patients, public and private healthcare providers, community health workers, test manufacturers, laboratory technicians, program managers and policymakers. Data were collected between January and June 2013 in rural and urban Karnataka, South India, as part of a larger project on barriers to point-of-care testing. We reconstructed patient diagnostic processes retrospectively and analyzed emerging themes and patterns.

**Results**: The journey to access diagnostic services requires a high level of involvement and immense work from patients and/or their caretakers. This process entails overcoming cost and distance, negotiating social relations, continuously making sense of their illness and diagnosis, producing and transporting samples, dealing with the social consequences of diagnosis, and returning results to the treating provider. The quality and content of interactions with providers were crucial for completion of test and treat loops. If the tasks became overwhelming, patients opted out, delayed being tested, switched providers and/or reverted to self-testing or self-treatment practices.

**Conclusion**: Our study demonstrated how difficult it can be for patients to complete diagnostic journeys and how the health system works as far as diagnostics are concerned. If new point-of-care tests are to be implemented successfully, policymakers, program officers and test developers need to find ways to ease patient navigation through diagnostic services.

## Background

Correct and timely diagnosis is essential for controlling infectious diseases such as tuberculosis (TB) and monitoring chronic conditions such as diabetes. Depending on a country’s specific diagnostic set-up, patients and providers play different roles in ensuring that correct and timely diagnosis is reached and treatment is initiated. India has a complex, fragmented healthcare delivery system, and in our previous work, we have shown that the onus is usually on the patient to ensure that test and treat cycles or diagnostic processes are completed [1,2]. This follow-up paper traces the diagnostic journeys of patients in South India and examines the work they have to do to arrive at a diagnosis.

Diagnosis of a disease condition is an integral part of medicine. Scholars studying the sociology of diagnosis have emphasized that the doctor and the diagnosis act as intermediaries in transforming illness to actual disease, and that in this process several dialogues and trade-offs occur [3,4]. According to Jutel [3, p. 278], ‘diagnosis takes place at a salient juncture between illness and disease, patient and doctor, complaint and explanation’ and ‘medical diagnoses are contested, socially created, framed and/or enacted’ [4, p. 794]. Such an understanding implies that a diagnosis does not exist in isolation, but is a concept that binds biological, technological, social and political aspects and the lived experience [3,4]. Thus, medical diagnosis is a composite activity and deserves consideration.

The majority of qualitative studies on diagnosis have focused on the health-seeking behavior of patients [5–9], but less so on the practicalities of the diagnostic system and the processes involved in reaching a diagnosis [10,11]. While some studies highlight different notions of risk, body, illness and healing that might influence decisions to seek a diagnosis [12], medical anthropologists such as Farmer [13] have emphasized that structural factors such as poverty and marginality determine care-seeking behavior and adherence to treatment or advice, rather than individual, rational decision making by patients.

While a lot of attention has been paid to challenges in seeking care, most studies have not focused enough on diagnostic processes, the use of diagnostic technologies, understanding of the structural factors and different actors’ perspectives in diagnostic processes. Accessing diagnostic services and arriving at a diagnosis require substantial work from the patient. As we will show in this paper, patients need to understand the disease condition, and have to make the decision to undergo a test, produce samples, collect the results and consult the treating physician again. Diagnostic work does not end with the initial diagnosis of a disease, but continues throughout treatment pathways with follow-up tests and monitoring of the disease. Unfortunately, patients are often absent in the discourses on diagnostics by decision makers, funders and test developers. Yet, they are expected to play an active role in these processes. To analyze the work involved by patients to arrive at a diagnosis, we use the concept of visible/invisible work that was introduced by scholars of science and technology studies [14]. These authors have highlighted the dynamic interplay between the visible and the invisible work, calling for rigorous analysis of both. One point of departure is to ask what exactly is work, and to whom it might (or should) be visible or invisible [15].

Studying the work done by patients to arrive at a diagnosis requires paying attention to the patient from the point of view of the test, and examining questions such as: Do I agree to get tested? Do I go to the lab to provide my sample? Do I pick up the results? Which lab do I go to? Do I deliver the result back to the same doctor or to a different doctor? What challenges do these steps entail for patients? And what consequences do these steps and challenges have for treatment adherence and impact on health? Such complexity is rarely traced in detail. The few studies that have examined the actual work that the patients have to do while completing diagnostic processes and arriving at a treatment decision have shown that acquiring a diagnosis is the composite result of personal priorities, social forces, the actions of healthcare providers, and the location and availability of services. This is likely to influence patient-important outcomes [16,17] .

Thus, it is critical to examine what challenges patients face in completing diagnostic processes across different healthcare settings by ‘playing an active role as a “diagnostic agent”’[15, p. 276]. Therefore, we conducted this study with the aim of understanding patients’ participation in diagnostic services, by examining the type of work that patients do, and why, and how to arrive at a diagnosis.

## Methods

### Setting

The results presented in this paper are part of a larger research project that explored the barriers to point-of-care testing in India, reported previously [1]. Study methods have been described in detail elsewhere [2]. In brief, data were collected between January 2013 and June 2013 in Kadugondanahalli (KG halli), one of Bangalore’s 198 administrative units, and Tumkur, a rural district in Karnataka (India). KG halli (estimated population 44,500 individuals) has pluralistic healthcare services and includes an area classified as slum. There are two urban primary health centers (PHCs) providing free outpatient care, which are managed by the local government. The private health sector consists of over 32 private providers, practicing various systems of medicine; modern allopathic medicine and Indian traditional medicine, AYUSH (ayurveda, yoga, unani, sidda, homeopathy). These private health facilities range from clinics with a single doctor to 50–100-bed private hospitals. Community health workers (CHWs) are employed by a non-governmental organization (NGO) (*n* = 6) and link workers employed by government (*n* = 7) carry out outreach work to educate the community about symptoms for a range of basic illnesses and refer them to appropriate health facilities. Tumkur district (population of 2.7 million), one of 30 districts of Karnataka state, was the rural study site. Private providers (*n* = 424) range from informal providers to highly specialized ones. The public sector has one district hospital with a capacity of about 450 beds, nine sub-district hospitals with a capacity of 30 –100 beds and over 140 PHCs. All of these public health facilities offer outpatient care, outreach activities and inpatient facilities with in-house laboratory (lab) facilities (except for a few PHCs). Public health facilities provide care free of charge for people living below the poverty line.

### Data collection

We conducted a total of 78 semi-structured interviews and 13 focus group discussions (FGDs) across different settings of the healthcare system (home, community, clinic, peripheral lab, hospital) to investigate diagnostic practices with healthcare providers (doctors, nurses, specialists, traditional healers and informal providers), patients, CHWs, test manufacturers, lab technicians, program managers and policymakers. The interviews and discussions focused on the major diseases that respondents dealt with in their respective setting. Respondents were selected through purposive sampling to represent different healthcare settings. For this paper, we mainly drew on seven semi-structured interviews with TB, diabetic and typhoid patients, and three FGDs with TB (*n* = 4), diabetic (*n* = 6) and hypertensive patients (*n* = 9). Demographics and clinical features were self-reported by participants. Table 1 provides the profile of the respondents of the seven semi-structured interviews. We drew on the material from the rest of the project where necessary [1,2] (an additional 10 FGDs and 71 interviews).

**Table 1.** Patient characteristics.

Respondent number	Age (years)	Gender	Education	Occupation	Area
R1 (TB patient 1)	70	Female	Illiterate	Housewife	Urban
R2 (TB patient 2)	19	Female	Secondary education	Student	Urban
R3 (TB patient 3)	30	Male	Secondary education	Brick business	Rural
R4 (TB patient 4)	49	Female	Secondary education	Housewife	Urban
R5 (TB and DM patient 5)	65	Male	Illiterate	Not working	Rural
R6 (DM patient 1)	40	Male	Primary education	Auto driver	Urban
R7 (TY patient 1)	30	Male	Illiterate	Daily wage earner	Rural

TB, tuberculosis; DM, diabetes mellitus; TY, typhoid.

Patients were asked to describe the major challenges they face while accessing diagnostic services for their particular condition (TB, diabetes, typhoid or hypertension). In both the interviews and FGDs, the explanations for these challenges and related solutions were explored as much as possible. Patil (MP) (a public health scientist and physician) and Engel (NE) (a social scientist) jointly conducted the semi-structured interviews and FGDs. Interview and FGD guides were piloted and revised accordingly and then translated into a local language (Kannada). Depending on the preference of the participants, interviews were conducted in either English or Kannada. All interviews and discussions with patients were audio-recorded. In addition, the note taker documented the main points raised, non-verbal communication and description of the setting.

### Data coding and analysis

To maintain confidentiality, personal details of participants were removed and audio files were anonymized. We constructed a full retrospective history of patients’ diagnostic processes from the onset of symptoms to the moment of diagnosis/initiation of treatment. All interviews and FGDs were transcribed verbatim. Three professional transcribers were hired to transcribe interviews materials from the audio-recorded versions. Each transcription was cross-checked by one of the researchers who conducted the interviews. MP, NE and VY jointly developed a coding scheme based on themes emerging from the data and the research questions, which aimed to examine what challenges patients face in completing diagnostic processes across different healthcare settings in the Indian health system. Each transcript, along with the field summary notes, was coded using NVIVO software version 9 by MP, NE and VY. These codes were tested on a handful of interviews initially and were refined over time. Such coding helped us to identify the themes and sub-themes that emerged from the interviews [18]. Memos were written on the diagnostic challenges that patients face. Later, emerging themes were identified. Relationships between and across themes were analyzed across different diagnostic processes and settings [19]. To increase the internal validity of the analysis, the coding scheme, the memos and the emerging themes were regularly discussed among the authors. The analysis indicated several constraints that patients face while accessing diagnostic services at different stages: making sense of the illness and diagnosis; making a decision to seek care, overcoming cost and distance; challenges in producing test sample and collecting the lab report; deciding to act on the lab result to consult the treating provider; and negotiating social roles while they complete these tasks. The results section elaborates on these themes to explain patients’ experiences in accessing diagnostic services.

## Results

The results are presented as follows. First, we illustrate the challenges during a patient’s journey to diagnosis by providing a narrative of a patient. From there, we proceed to present the findings under the following major themes: (1) making sense of illness and diagnosis; (2) overcoming cost and distance; (3) producing and transporting samples; (4) collecting and acting on the results; (5) interacting with providers; and (6) negotiating social roles.

**Figure 1.**
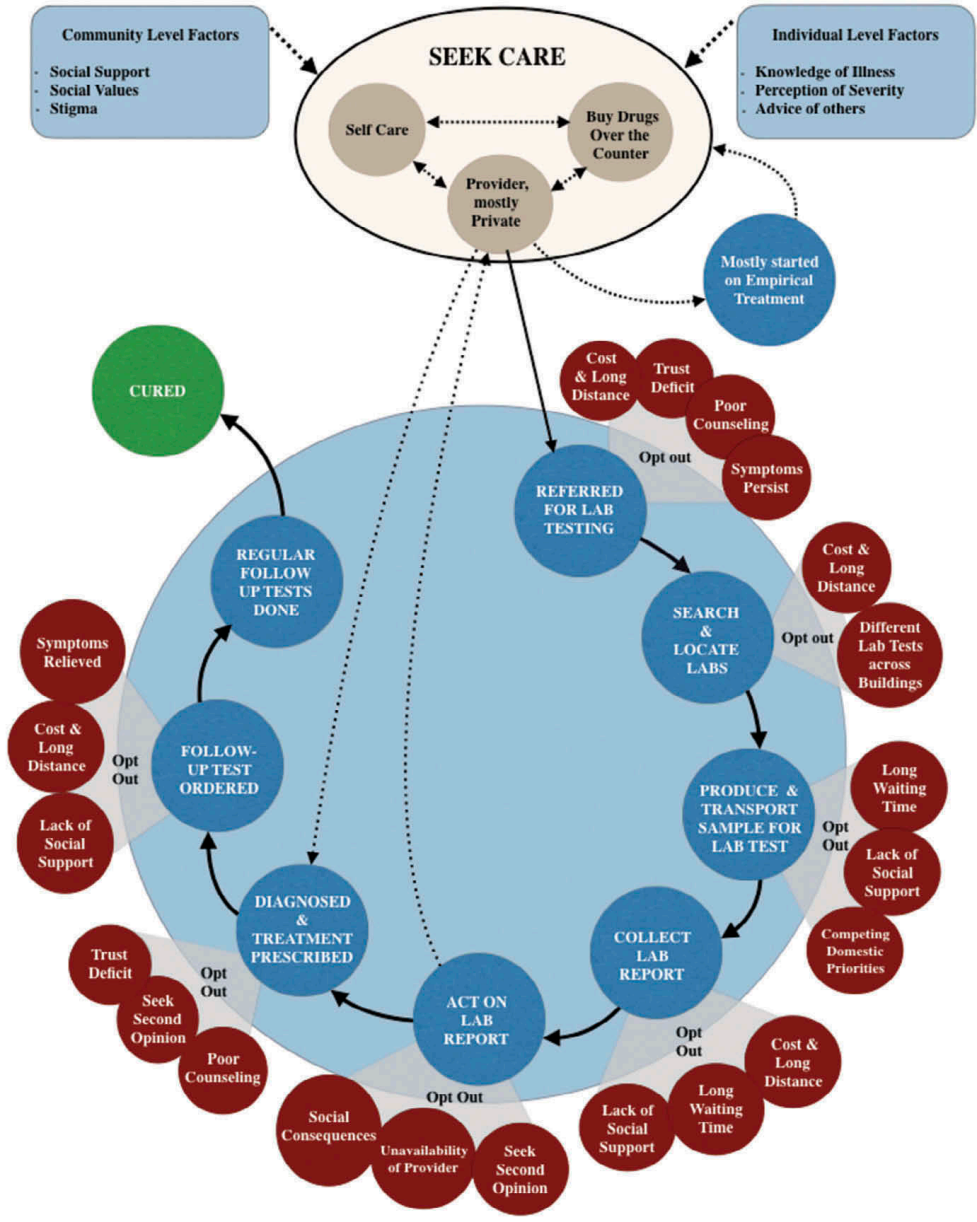
Patient navigation in the diagnostic ecosystem.

Patients have to occupy a central role in the Indian healthcare system to ensure a successful diagnosis. When patients need to have a diagnostic test done, they have to find a lab, go there to provide a sample, return when the results are available, pay for the service and return to their doctor with the results. Patients carry their samples, reports and communication between providers across all settings, small private clinics as well as large public hospitals. In doing so, they need to navigate the complex and fragmented health system, and go back and forth between a variety of public and private providers at different levels of care. The particular Indian context has important consequences for the work that patients have to do to arrive at a diagnosis. In doing so, they need time, money, patience, social support and resilience, and if these are lacking, patients tend to opt out of the diagnostic cycle. In Figure 1, we illustrate some of these challenges during a patient’s journey to diagnosis, and we also provide a narrative of a patient below (R4, TB patient).

A woman aged 49 years, living in a poor urban area, started having a cough, fever and backache. She self-medicated at home for a month, buying medicines from a local private pharmacy. When the symptoms became unbearable, she visited a public clinic where she was asked to undergo a sputum test to rule out TB. Since she could not bring out a sputum sample on the spot, she went home, but then decided not to return to the clinic. Her symptoms worsened and she went to a nearby private clinic, where she was treated with injections, tablets and intravenous fluids for 10 days. Since her symptoms did not subside, she was asked to take a blood test to rule out typhoid. The test turned out to be positive and she took typhoid treatment for the next 20 days. Yet, the symptoms did not subside. Her daughter took her to another private clinic nearby, where she was treated with tablets and injections for a week with no success. Instead, her condition deteriorated. Later, her husband took her in an auto-rickshaw to a nearby private secondary care hospital, where a series of blood and urine tests was done in the in-house lab, followed by a chest X-ray (cost: USD 5). Her husband collected the reports from the in-house lab the next day and showed them to doctor, who advised him that his wife should be admitted to hospital. That evening, the doctor removed the fluid that had accumulated in the back of her lungs, based on the image of the chest X-ray. Her husband transported the fluid to a big private lab for further tests (USD 6) and collected the report the next day. The doctor confirmed that she had TB. Three days after being admitted, she was advised to take free TB treatment from the government hospital. At the nearby government hospital (in X-nagar), she was told to access another government hospital (Y halli), where she was again asked to go to another government hospital (Z halli), close to her home. She traveled by auto-rickshaw, but here treatment was also denied because the results were from a private lab. Hence, she was referred to a medical college hospital to obtain another diagnosis. After traveling another mile by auto-rickshaw, the patient underwent sputum, urine, blood tests and chest X-ray in the in-house lab of the medical college hospital. Her husband collected the lab reports the next day and showed them to the same doctor in the medical college, who advised that the patient be admitted to the hospital for a week. During this time, fluid was removed from her back twice and TB treatment was started in the TB center located within the hospital. She was discharged after a week and was referred to a public clinic in her vicinity to continue TB treatment. She went for a follow-up every month to the medical college hospital, as advised by the physician there. She was asked to obtain a computed tomography (CT) scan from a private lab, as these were not available in the medical college hospital (USD 60). When she returned to the physician at the medical college hospital, he confirmed that no other parts of the body were affected by TB. During the follow-up visits, another chest X-ray was taken to check whether the fluid in her back had reduced. She was advised to continue TB treatment for 6 months. She collected the tablets from a nearby public clinic and went to the medical college hospital for follow-up chest X-ray once a month, accompanied by her daughter, and completed the treatment. She estimated the total cost of the diagnosis, treatment and transportation to be around USD 602.

This example is illustrative of some of the main challenges that patients encounter while accessing diagnostic services. It also shows that accessing care may be the first step towards a diagnosis, but patients have to continue seeking further care and follow-up testing according to referral instructions, reappearing symptoms and treatment protocols. Therefore, a diagnostic journey is not necessarily over once a final diagnosis has been acquired and the disease has been established.

In the following sections, we describe the themes that emerged from the data: making sense of illness and diagnosis; overcoming cost and distance to arrive at a diagnosis; producing and transporting samples for lab tests; collecting and acting on the results; interacting with providers; and negotiating social roles. We draw on examples from patients struggling with different diseases, such as TB, diabetes, hypertension and typhoid.

### Making sense of illness and diagnosis

The decision to seek care from a provider and overcome associated challenges of cost and distance required a constant, active sense-making of the illness and diagnosis. This active sense-making was shaped by the patients’ own explanations of diseases and their causes, trust established with providers, and perceived quality of care by patients and their family members. A TB patient explains how he relied on his neighbors’ or his family members’ earlier experiences to decide where to seek care:

Our neighbors will advise ‘we had been there and it has been cured, you too go there’. Then we go there. This time, [the reason] why we went there [private hospital] is my sister told my son ‘do not go here, just take her [patient] there. They will tell you everything’ and then they [family members] took me there. And after we went there, I came to know that I had TB. (R4, TB patient, urban)

Patients’ own explanations of illness influence how and when they seek care. The diabetes and TB patients in this study thought that the initial symptoms were due to the common cold, cough, fever or dust allergy (for TB); and tiredness, excessive hunger and thirst were attributed to normal tiredness due to hard work (for diabetes). Hence, they resorted to self-medication practices in the initial stages of the illness. They purchased medicines without a doctor’s prescription from a local pharmacy over the counter for varying periods ranging from a few days to a month. When the symptoms did not reduce with these medications, they decided to seek care from a doctor. A quote from a TB patient illustrates this:

Up to one month, I did not go to any hospital, neither did I talk to anyone anything about this. My son used to get tablets from nearby pharmacy shop and give it to me. I would take it and sleep. In the morning I would do my work as usual. But by then, I became very lean. (R1, TB patient, urban)

Some patients avoided seeking care because of the fear of undergoing invasive procedures. According to the CHWs we spoke to, some patients tend to seek care from religious practices first, especially for neurological disorders, TB and human immunodeficiency virus (HIV), which are thought to be caused by the devil and thus require divine intervention to cure them (FGD 3, Accredited social health activists). To quote from a patient from the FGD with TB patients:

We were scared of the tests and we believed that it would reduce by divine intervention. People say that making a wish to God will help. So we tried that method too. But it was of no use. So we got the test done. Only after the test was done it [TB] was confirmed. (FGD 5, TB patients)

Patients suffering from hypertension to whom we spoke go to the government facility, free of charge, for regular follow-up tests, but prefer to buy medicines from a private pharmacy. This is because they perceived the potency of drugs available at the government hospital to be weak. An FGD participant explains:

I go there [government hospital] for check-ups, but I take tablets from the private chemist shop. Because the tablets given at the government hospital is not improving my condition. So I buy it outside. (FGD 10, Diabetes and hypertension patients)

Patients sometimes insist on blood tests during their first visit to a provider, as they believe that just by having their blood tested they might be cured, irrespective of the treatment. A lab technician working in a private lab narrates how a patient who was referred from a private provider for lab tests complained that he was not referred for a blood test at the first instance:

For many people, just a blood test or just touching them is enough to cure them. More than the doctor’s treatment, this kind of psychology exists with patients. I did a blood test just like that without reason to a patient. Earlier to this, that patient had been to a doctor for a week and the doctor sent back the patient without referring for tests. So patient told [me], ‘I told him [doctor] on the first day to order a blood test but, he said it was not required. Now see after I got it [blood test] done, I am cured’ … there are many such examples. (Lab technician 15)

### Overcoming cost and distance

We found that cost and the distance to the labs or health facilities are important factors determining patients’ diagnostic journeys. The majority of patients preferred to seek care from private providers because of the perceived good quality of care there. Solo private clinics providing primary care are usually accessed at the first instance by patients with low income. Private secondary care hospitals are mostly accessed by the middle class, while private tertiary care hospitals are mostly used by the highest income class.

After unsuccessful self-medication, patients from lower socio-economic strata seek care at the nearest health facility irrespective of whether the provider is qualified or not. In one such case, the father of a 4-year-old typhoid patient spent around USD 10 per day for seeking care from private providers, whereas his daily wage was USD 3. Patients suffering from chronic illness, such as diabetes, were aware of the fact that they should monitor their blood glucose levels regularly and go for follow-up visits. However, they were unable to access continuous care, because the government health facilities providing free services either were too far away or did not provide these services, or patients had to pay informal fees for consultation and lab tests there (FGD, CHWs). Therefore, they were forced to go to private labs for follow-up tests, but patients found it difficult to afford the fees in the long run. Others traveled to a charitable hospital situated away from their residences, where blood sugar testing and medicines were provided at subsidized rates (R6, Diabetic patient 1, and FGD 4, Diabetes patients). However, the long distance, transportation costs involved and loss of wages prevented patients from regularly going for follow-up visits to monitor their blood sugar levels.

They have told me to come regularly for check-ups. It has been 3 months since I went there. I do not have money now. So, I am not going. Previously, I used to go once in 6 months or once in 3 months. It all depends on my financial condition. If I have money, I will go or else … The blood test costs 50 rupees, 10 rupees to buy the OPD card there and expenses for traveling by bus, to and fro, it comes to about 100 to 150 rupees for one visit. (FGD 4, Diabetic patients)

The high cost of testing and long distances to testing centers meant that patients delayed being tested, switched providers, and/or reverted to self-testing and self-treatment practices. There were various ways in which patients, especially those suffering from chronic illnesses like diabetes, coped with monitoring their disease. For instance, some diabetic patients self-monitored their blood glucose levels by intuitively recognizing the symptoms associated with a rise in their blood sugar level, such as feeling tired, increased aches and giddiness. Others self-medicated based on a diagnosis and old prescriptions given 2 or 3 years ago, and adjusted the dose when they felt that their blood sugar had increased. Finally, some patients went directly to the private labs for blood sugar tests to save the doctor’s consultation fee. Because of these compromises, their blood sugar levels were usually high and only when symptoms became unbearable did patients seek care. For example:

It has been 2 years since I had been to X Hospital [government diabetic research institute]. But now even to go there, I do not have any money. If I go to Dr Y [private practitioner] for a check-up, there we have to give money. They had told me, if the sugar increases. Instead of one tablet I have to take one-and-a-half tablets. I am trying to adjust by taking the tablets in that way. (FGD 4, Diabetic patients)

With these financial and transportation constraints, patients had to make tough trade-offs between seeking care and other immediate demands. Often this meant prioritizing domestic needs over health needs. A patient narrates how she has been managing these conflicting priorities:

We have to go by walk[ing] till the police station to reach that government hospital. There are no short-cuts to reach there. The main problem in this locality is water. We have to collect water in pots and bring it home. Then we have to go and dispose [of] the garbage … if I have to walk up till there [public hospital], how can I do these works? So we decide to pay money and get it done nearby when we have money and when we do not have any money, we will just not go at all. (FGD 4, Diabetic patients)

Thus, in many instances, poor patients were not able to afford any lab tests. Instead, they preferred a prescription for drugs or directly purchased over-the-counter drugs and completely bypassed the medical consultation process to save money. Patients and families had no room for price negotiation with either providers or lab technicians and felt powerless to meaningfully shape the healthcare they received. Hence, many patients either decided not to go back to the same provider if they were not able to follow the provider’s advice and/or reverted to self-medication.

### Producing and transporting samples

After the clinical examination, providers tended to make a provisional diagnosis and order tests to confirm the diagnosis. We found that producing a sample for lab tests requires considerable work by patients and their families. This work is not always easy and costs time and money, can have social consequences, and may mean that patients opt out and seek care from other providers or self-medicate. A patient with chest symptoms may have difficulties in producing a sputum sample to test for TB by coughing loudly during a consultation in a government clinic. One of the female respondents (R4, TB patient 4) felt awkward coughing loudly to bring out the sputum in the middle of the waiting room. Hence, she was asked to collect the sample at home instead. According to her, the symptoms did not subside, so she decided to switch provider instead of providing the sample and sought care at another nearby private provider.

First, I went there [PHC]. There, the senior doctor said, ‘you have some problem, you give a little phlegm. I will check it and tell you’. I tried a lot, but the phlegm did not come. I told the doctor ‘it is not coming’ and she said ‘bring it tomorrow morning’. I just came back and never went there again. (R4, TB patient, urban)

In contrast, CHWs suggested that making the choice to switch providers was due not so much to the inability to produce the sample or the fact that symptoms did not subside, but to the lack of privacy to produce a sample at home. Because of the stigma surrounding TB and the violent coughing involved in producing the sputum, patients feel uncomfortable in producing sputum samples, even when at home, especially when there is a chance of their neighbors watching or hearing their coughing.

In many cases, producing a sample not only was difficult, but also demanded time and money from patients, and had social consequences. Patients who were diagnosed with a disease in the private sector, but sought care in the public sector to access free drugs, had to undergo a repeat lab test to be eligible for free drugs. Results obtained from private labs were not accepted by the public sector health facilities, and they tended to repeat all of the required lab tests, depending on the patient’s symptoms. Repeating a test involves work from patients and their caretakers, such as queuing in the lab, producing the sample, collecting the lab report and returning to the treating provider. Different tests were often done in different buildings. For instance, it was mandatory for all TB patients diagnosed in the National TB program to undergo an HIV test, which was done in another building situated half a kilometer away. Each time, the patient had to wait in different queues to be tested, produce samples, collect separate reports from different places at different indicated times (often the next day) and queue again to see a doctor. Similarly, diabetic patients reaching public health facilities end up roaming across three different sites to be tested.

In the same vein, patients undergoing diagnosis for extrapulmonary TB, for instance TB of lymph nodes or pleural TB, relied on family members or hospital staff to transport samples to bigger labs for testing. Depending on the organ affected, different tests such as fine-needle aspiration cytology, pleural tapping for fluid analysis or scanning were ordered. Many of these tests were not available in the in-house labs of secondary care hospitals, either private or public. In rural community settings, government field health workers and local NGO staff had the responsibility of collecting patients’ sputum samples and transporting them to the microscopy center of nearby public health facilities.

### Collecting and acting on the results

The onus of collecting the lab report and taking the results back to the treating doctor was entirely on the patients and/or their relatives in all settings, and involved substantial time and cost. In urban hospitals, it took, on average, 12 hours to for outpatient department patients to receive their lab results. If the samples were provided in the afternoon or evening, the patients were expected to come back the next day to collect the results. In contrast, urban private tertiary care hospitals had a different mechanism, where outpatients provided their samples at a lab reception situated next to the consultation rooms and collected the reports from the same reception. Although the turnaround time for collecting the results remained the same, this counter helped the patients and their caretakers to navigate the collection of reports more easily because they did not have to pick up results for different tests, e.g. TB and HIV examinations, from different labs. For patients admitted to the tertiary hospitals, lab results were sent directly to the wards through a computer system, which the staff nurses checked and then informed the treating doctors of the results (Lab manager 1). In small hospital settings, either the patient’s relatives or nurses collected the lab results.

In rural areas, patients who sought care in sub-district or district public hospitals often had to return to their village in the evening. Test results were only made available on the next day. Therefore, some could not afford to wait that long, especially daily-wage workers (whose average daily income is around 4.2 USD). This dissuaded them from collecting results or led to arguments with lab staff over expediting the process (Specialist 2). Most of the diabetic patients in the urban area preferred to go to one of the many smaller private providers who offer blood sugar testing during the consultation and provide immediate results. This saved time for these patients (FGD 4, Diabetic patients). Alternatively, patients made adjustments to their working hours to align with the working hours of providers. They provided blood samples in the morning at small private labs, and collected lab results that same evening on their way back from work to consult the treating providers. Sometimes after consultation with a doctor, patients decided not to engage in the work of having the lab tests done and/or decided to switch providers. This happened when patients could not afford the costs of investigations, or when they expected that symptoms would improve with the provider’s medication and they would not have to spend money on unnecessary lab tests, or when symptoms did not reduce after a few days.

### Interacting with providers

In the process of obtaining a diagnosis, patients and their relatives interact with a variety of healthcare providers, including doctors, CHWs, lab technicians and staff in the clinic and hospital administration. The quality and content of these interactions had implications for the quality of the samples produced, the diagnostic results and treatment initiation, and thus the patients’ health outcomes. Established trust with a so-called ‘family doctor’ played a crucial role in patients navigating the diagnostic pathway. These doctors were often consulted for primary care and patients preferred to seek care there, as the doctors were very familiar to them (TB patient 3 and Diabetic patient 1). They did not seem to mind spending money and losing a day’s earnings, and took out loans to pay for the expenses when they visited their family doctors (FGD 4, Diabetic patients). One patient explained why he trusts his family doctor and prefers to go there, irrespective of the cost:

Not because it is cheaper there, he definitely charges fees. We go there because he treats us well. We are confident about the doctor. (R3, TB patient, rural)

In general, patients were anxious to know about the possible diagnosis and treatment, but the majority of the doctors did not find time to explain to patients the disease course and diagnostic tests required. Providers did not inform patients about the lab tests they had ordered, to circumvent discussing their suspicions, especially in case of TB or HIV, which are stigmatized diseases. Instead, they wrote a referral slip and referred patients to labs and discussed the disease only when the lab results turned out to be positive. Providers believed that patients do not have enough knowledge about the diseases (private providers PP2 and PP5) and preferred to keep patients uninformed to avoid scaring patients away (PP5), and to protect themselves if their provisional diagnosis was wrong. This was reiterated by a TB patient:

I consulted so many doctors. Took so many medicines. But nobody told me that it was this disease [TB]. That is why I am still angry with those doctors. Everybody said that a blood test will reveal what the disease is. That doctor did it [blood test] for three times, not once did he tell me why I got this disease [TB]. Finally, at the last moment, when I was absolutely unable to move and very tired, I went to this [private] hospital and doctor said water has filled up and removed the water from [my] back. (R4, TB patient, urban)

We observed that patients’ sense-making of illness changed if clear explanations were given for the causes of the disease. For instance, a patient did not accept the provisional diagnosis of diabetes made by a doctor, because he perceived diabetes to be a major disease. So, he sought a second opinion from his family doctor, who explained the causes, types and symptoms of diabetes, which altered the patient’s own explanation of the severity of the illness and helped him to cope with the disease (Diabetes patient 1). It appeared that when a disease changes from a ‘major’ to a ‘common’ disease, the stigma attached to it lessens and patients tend to seek a diagnosis more readily. To quote one patient:

Now TB is a common disease, it is not dangerous like in those days. TB disease is just like a fever now. In order to get it cured, we got it tested and came here. (FGD 5, TB patients)

In another instance, a patient presented with a lump in her neck. Her relatives delayed having the investigations done, for fear of her being diagnosed with cancer, which they perceived to be grave. Since they did not receive a satisfactory reply from the provider, the patient’s relatives lost trust in the provider and subsequently kept switching providers in the anticipation of a less severe diagnosis. Finally, when a private provider ordered a lab test, it turned out to be extrapulmonary TB. The patient and her relatives were relieved to learn, through the counseling provided by the treating physician, that the lump was caused by TB and not cancer, and that it could be cured with 6 months of treatment. One of her parents said:

My daughter had a throat problem since 5 to 6 months. We took her to private doctors and also to the government hospital. There they said that tests needed to be done. We got scared after hearing them and did not get it done anywhere. We thought it could be cancer. (FGD 5, TB patients)

Some providers were offended when patients asked questions about the diseases they had been labeled with, because the providers considered that patients were not qualified to question their authority. Some of the diabetes patients knew that blood sugar testing was available at public facilities, yet did not dare to ask about its availability, thinking that questioning the provider is equal to disobedience:

Even if the diabetes testing machine is there [at the public clinic], they [staff] will say, ‘It is not there’. Once I asked them, whether they would do the test. They replied to me that, they do not do any test. So I just left it at that. If we ask like that, we will be scolded. (FGD 4, Diabetic patients)

These results highlight that a thorough explanation of the disease specifics by the provider and the need for lab tests increase the likelihood of undergoing diagnostic tests (TB and DM patient 5). A diabetic patient, for instance, ignored the lab test ordered by a provider who simply voiced the suspicion of diabetes. The patient thought that the provider was lying, and sought care from another private doctor nearby. This second doctor explained the different types of diabetes, conducted a blood test (with a glucometer) in front of the patient and referred him, with a reference slip, to a nearby private lab for confirmatory tests. As the quote below shows, the patient appreciated the counseling provided by the second provider as he was able to link his symptoms to the suspected disease, and decided to have the lab test done:

Only later when the other doctor explained, I came to know that excessive sleep, getting tired, passing urine frequently are symptoms of diabetes. In diabetes, there are three to four types. Then I decided to get my blood checked. (R6, Diabetic patient, urban)

### Negotiating social relations

In following diagnostic pathways, patients had to negotiate various social relations to ensure support. Elderly patients and women were often dependent on family members’ help to travel to health facilities, and had to ask permission to spend money on care, to have lab tests done or even to go for follow-up visits. One of the TB patients relied on his son to travel to the hospital by bus for half an hour. Without this support, he could not have gone for any follow-up tests during the 6 months of TB treatment (TB patient 5). In poor urban areas, women were restricted to their homes and had limited autonomy in seeking care at health facilities or advice from CHWs. They had to negotiate not only with their husbands, but also with their mothers-in-law, to seek care. The CHWs in urban areas recalled several instances where mothers-in-law prevented their daughters-in-law from having prenatal check-ups as per the CHWs’ advice, stating that they themselves had had several deliveries without undergoing any lab tests (FGD 7, Link workers, and FGD 8, Auxiliary nurse midwives).

Negotiating such crucial social support was particularly challenging in the context of stigmatized diseases such as TB. The majority of TB patients interviewed had not revealed their disease to anybody except their family members, fearing discrimination and ill-treatment from neighbors. A diagnosis of TB could also lead to conflicts within the family. In one such case, a husband was angry with his wife for being diagnosed with TB, because TB had never occurred in their family before (TB patient 4). The implications are that patients delayed seeking care and found it difficult to negotiate social support to continue diagnostic and treatment follow-ups.

## Discussion

Our study demonstrates that patients’ journeys in accessing diagnostic services involve immense work by patients to make the diagnostics work. Our results show that in the Indian context, patients often move between providers and across different levels of care, amassing diagnostic delays, frustration and ambiguity. If the tasks become overwhelming, patients either opt out, delay being tested, switch providers and/or revert to self-testing or self-treatment practices. The work involved for patients in acquiring a diagnosis and following through diagnostic pathways and treatment protocols entails continuously making sense of illnesses and diagnosis, overcoming cost and distance, producing and transporting samples, collecting and returning results to the treating doctor, negotiating social relations and dealing with the social consequences of diagnosis. For this to work effectively, patients have to actively make sense of the illness and diagnosis, need to have enough knowledge to understand that they need to be examined and treated, and need to know where in the health system this is offered. In doing so, patients are challenged by their own socio-economic constraints, competing domestic priorities, poor transport and absent health facilities, and the reliance on social support networks to access care and collect test results. The quality, content and dimensions of interactions and relations with healthcare providers are crucial at each step, with direct implications for the quality of results and the completion of test and treat loops.

Our study confirmed the larger theoretical issue highlighted by Strauss and colleagues that clients of services, in our case patients as the recipients of diagnostic services, become part of the division of labor that is required to complete the work of diagnosing. All the while, neither the provider nor the patients may recognize their efforts as actual work [17]. A patient’s hard work to achieve a diagnosis is often not recognized and appreciated by the providers, test developers, funders and decision makers, because this work is largely invisible to them and it is assumed that patients automatically follow the providers’ advice. Patients in India perform a kind of bricolage to successfully complete the diagnostic process, by adapting practices, and integrating practicalities, their own knowledge, expectations and explanations of illnesses and the medical system, social relations, and the tasks that are asked of them. This work is a constant and fluctuating process, but sometimes aligning these elements is too difficult and thus patients may opt out. Opting out of the health system does not mean that patients necessarily stop seeking care; they may revert to self-medication (diabetes) and learn to ‘manage their illnesses’ with or without involving professional help [16]. Thus, patients take an active part in the medical work. In the following, we discuss some of these results in more detail.

Following through diagnostic journeys costs patients valuable money and time. This becomes a bigger issue if testing requires several visits or needs to be accessed across different labs in a hospital campus or at different levels of care, or if unnecessary or wrong tests are ordered. For instance, the inability of private providers to diagnose TB was reported by almost all the TB patients interviewed in this study. Private providers ordered lab tests that were not specific to TB, a finding recently confirmed by a ‘mystery clients’ study [20]. This made the patient’s pathway to TB care a complex and costly experience. In this process, patients lost trust in services provided. In addition, TB patients whom we interviewed had to make at least three visits to public health facilities to obtain their sputum results. A study from India showed that a distance of more than 10 km to the diagnostic facility was associated with loss to follow-up among those with TB symptoms [21]. This points to the need to develop point-of-care testing programs and ensure that patients can obtain results within one visit.

The work that is done by patients involves not only the literal tasks of going to the health facility, providing samples, returning with results, queuing and waiting, etc., but also continuously reframing notions of what needs to be done. If this reframing does not take place, it does not make sense to invest the necessary effort, time or money to make the diagnostic journeys worthwhile. The narratives in our study highlight that the burden of undergoing tests and collecting reports almost always falls on the patients and/or their relatives. This is complicated by the fact that patients often switch between different providers, amassing delays. In the public system, for instance, referrals often do not work or still cost money, and patients may end up losing even more money on expensive investigations and treatment in private hospitals. In this case, the sense-making work to follow through diagnostic pathways breaks down and it makes more sense to seek explanations, comfort and cure elsewhere (e.g. by going to the temple).

In seeking diagnostic services, patients also had to negotiate various social relations. These results confirm earlier studies showing that sociocultural determinants such as gender (being female), the status of married woman and occupation reported as housewife have been associated with diagnostic delays [22,23]. Husbands may become upset when their wives are asked to undergo testing for HIV, since it is associated with immoral behavior [24]. Similarly to our study findings, other studies have reported that female patients tend to receive less support from their families and often face financial, physical and social barriers in accessing health services [25,26]. Our results show that stigma and anticipated social consequences are factors that cause delays in seeking a diagnosis. The stigma associated with diseases such as TB is highly prevalent in India [27], with consequences such as discrimination and loss of status [28]. However, we saw that stigma plays less of a role if patients fear a potentially fatal diagnosis of a disease such as cancer, which is thought to be more serious than TB.

Studies have shown that thorough explanation of the testing processes, the testing venue and knowing what needs to be done after the receipt of lab results increase the likelihood of undergoing a test, especially for stigmatized diseases [9]. If, on the contrary, patients believe that providers are withholding information, this can lead to mistrust, which can hamper the willingness to undergo tests and access care [29]. The relationship between doctors and patients is often marred by mistrust and poor communication [30]. Doctors are offended when patients ask questions or seek a second opinion [31–33], and do not sufficiently encourage clients to be tested for HIV after their initial decline [7]. Rather, they keep patients uninformed about a provisional diagnosis, particularly in the case of stigmatized diseases. Therefore, instead of defining non-adherence to the advice of doctors as a medical problem due to deviant behavior by patients, it has long been suggested that the focus should be on the importance of communication in the patient–provider relationship [34]. Yet, our results show that providers often did not explain to patients the need for a particular diagnostic test, the specific testing procedures and what the results mean. Patients in our study were expected to automatically follow the doctor’s testing decisions or advice. As noted by Barbot [35, p. 539], ‘providers expect patients “to co-operate” in order to acquire the cognitive and moral references of their medical environment, and thereby usefully participate in their own therapy’ [35]. A study on medical encounters in TB care in India showed that healthcare providers adopted an authoritarian approach to persuade ‘the ignorant patient’ to follow their advice [36]. Therefore, building a trust-based relationship between providers and patients through taking the time to explain and communicate the different aspects of diagnostic tests, procedures and possible diseases is vital in making sure that patients follow the advice to undergo tests.

## Conclusion

Our study demonstrates the various challenges that patients have to overcome to follow through diagnostic pathways and thus to ensure completion of ‘test and treat’ loops. If new diagnostics and point-of-care testing are to be implemented more widely, these barriers must be addressed and more research should be conducted that can guide adoption and scale-up in real-world settings. Our study reveals not only patients’ perspective on diagnosis and seeking care, but also how the health system works as far as diagnostics are concerned. This study should also demonstrate to the medical profession how difficult it can be for patients to complete these journeys, and remind policymakers, test developers and program officers to find ways to ease patient navigation through diagnostic services.

## Paper context

Ensuring timely diagnosis and linkages to care at the point of care in India is challenged by limited infrastructure, money and human resources; diverse non-coordinated providers; and dominant symptomatic treatment. This paper details the immense work that patients need to do to complete their diagnostic journeys in this fragmented health system, which includes continuous sense-making and negotiation of social roles. The results reveal the key role of the relation with health providers in easing these journeys.
